# Breast Cancer Metastasis Suppressor 1 Regulates Hepatocellular Carcinoma Cell Apoptosis via Suppressing Osteopontin Expression

**DOI:** 10.1371/journal.pone.0042976

**Published:** 2012-08-21

**Authors:** Yanhua Wu, Wenjun Jiang, Yingming Wang, Jun Wu, Hexige Saiyin, Xiaojing Qiao, Xinyu Mei, Bin Guo, Xiao Fang, Lu Zhang, Huiling Lou, Chaoqun Wu, Shouyi Qiao

**Affiliations:** State Key Laboratory of Genetic Engineering, Institute of Genetics, School of Life Sciences, Fudan University, Shanghai, People's Republic of China; University of Hong Kong, Hong Kong

## Abstract

Breast cancer metastasis suppressor 1 (BRMS1) was originally identified as an active metastasis suppressor in human breast cancer. Loss of BRMS1 expression correlates with tumor progression, and BRMS1 suppresses several steps required for tumor metastasis. However, the role of BRMS1 in hepatocellular carcinoma (HCC) remains elusive. In this study, we found that the expression level of BRMS1 was significantly down-regulated in HCC tissues. Expression of BRMS1 in SK-Hep1 cells did not affect cell growth under normal culture conditions, but sensitized cells to apoptosis induced by serum deprivation or anoikis. Consistently, knockdown of endogenous BRMS1 expression in Hep3B cells suppressed cell apoptosis. We identified that BRMS1 suppresses osteopontin (OPN) expression in HCC cells and that there is a negative correlation between *BRMS1* and *OPN* mRNA expression in HCC tissues. Moreover, knockdown of endogenous OPN expression reversed the anti-apoptosis effect achieved by knockdown of BRMS1. Taken together, our results show that BRMS1 sensitizes HCC cells to apoptosis through suppressing OPN expression, suggesting a potential role of BRMS1 in regulating HCC apoptosis and metastasis.

## Introduction

Hepatocellular carcinoma (HCC) is one of the most common cancers worldwide, with high prevalence in parts of Asia and Africa, and incidence is increasing in western countries. While standard chemotherapy and radiotherapy have limited efficacy in most HCC patients, surgical resection and liver transplantation remain the current curative options to treat HCC. However, HCC is highly associated with invasion and metastasis, which leads to poor prognosis after surgical resection. To develop effective therapeutic targets for HCC, it is essential to gain further insight into the molecular mechanism involved in HCC progression, especially in HCC metastasis.

Metastasis suppressor genes are a growing family of molecules that inhibit metastasis at any step of the metastatic cascade without affecting primary tumor growth. To date, approximately 23 metastasis suppressors have been identified, including Nm23, Kail and E-cadherin, among others, representing a potential group of prognostic markers and therapeutic targets for tumor metastasis [1]. Breast cancer metastasis suppressor 1 (BRMS1) is a metastasis suppressor that was first identified in breast cancer [2], [3]. BRMS1 has since been shown to suppress metastasis in many other cancer types such as melanoma, ovarian carcinoma and non-small lung cancer, among others [4], [5], [6], [7], [8]. BRMS1 expression in these tumor tissues is negatively correlated with tumor progression and metastasis [7], [9], [10], [11], [12]. Mechanistically, as a component of the mSin3-HDAC complex, BRMS1 regulates chromatin status and therefore modulates the expression of genes functioning in cell apoptosis, cell-cell communication and cell migration [13], [14], [15], [16]. Osteopontin (OPN), also known as secreted phosphoprotein 1 (SPP1) and originally identified in osteoblasts, is transcriptionally regulated by BRMS1 in breast cancer [17]. Extensive studies have demonstrated that OPN is an important regulator of tumor metastasis, with multiple roles in cell adhesion, cell migration, cell survival, tumor angiogenesis and other activities [reviewed in [18]]. It was found that, particularly in HCC, exogenous OPN expression made non-metastatic HCC cells more invasive *in vitro* while knockdown of endogenous OPN expression or blocking OPN function suppressed *in vitro* cell invasion and *in vivo* tumor metastasis [19], [20], [21]. Clinical studies further revealed that elevated expression of OPN is associated with advanced tumor grade and tumor stage, vascular or bile duct invasion and intrahepatic metastasis [22], [23], [24], [25]. Both tissue and serum OPN levels were demonstrated to be predictors of HCC recurrence and poor prognosis [26], [27], [28].

Despite the current data, neither the role of BRMS1 nor the relationship between BRMS1 and OPN in HCC has been investigated. By using HCC samples and pair-wise, non-tumorous liver tissues, we found a remarkable down-regulated expression pattern of BRMS1 during tumor progression, indicating the involvement of BRMS1 in HCC. Expression of BRMS1 sensitized HCC cells to apoptosis, whereas knockdown of endogenous BRMS1 protected cells from cell death. OPN expression was suppressed by BRMS1 in HCC cells, and knockdown of OPN rescued the anti-apoptosis effect induced by BRMS1 knockdown.

## Results

### BRMS1 was significantly down-regulated in HCC tissues

**Figure 1:**
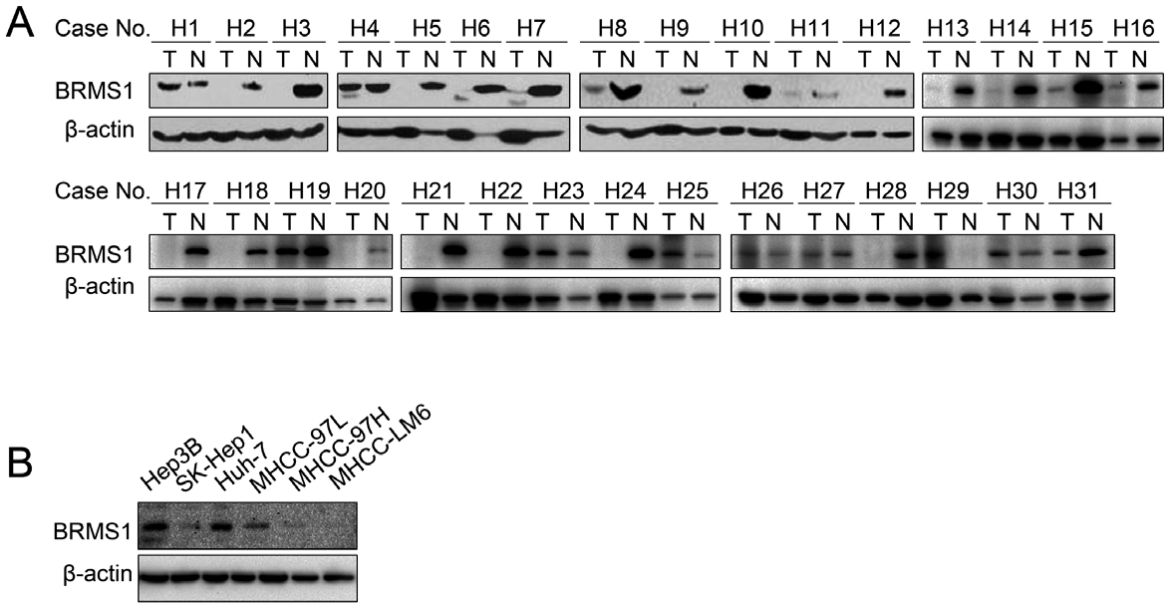
BRMS1 expression pattern in HCC tissues and cells. (A) The expression level of BRMS1 was analyzed in HCC tissues (T) and corresponding non-tumorous tissues (N) by western blotting analysis using an anti-BRMS1 antibody. β-actin was used as a loading control. (B) The expression level of BRMS1 in different HCC cells was also investigated through western blotting analysis.

To elucidate the role of BRMS1 in HCC, we first detected BRMS1 expression in HCC tissues. Thirty-one paired HCC tissues (T) and their corresponding non-tumorous tissues (N) were utilized in western blotting analyses using a specific anti-BRMS1 antibody. β-actin was immunoblotted as an internal control. As shown in Fig. 1A, the BRMS1 protein level was remarkably suppressed in 19 HCC tissues compared with their corresponding normal tissues. No significant differences were shown in ten cases, and BRMS1 was up-regulated in the other two cases. A significant difference in BRMS1 expression was revealed between tumor and corresponding normal tissues (*P = *4.75E-05, paired Student's *t* test). Among all of the specimens, clinico-pathological data from 21 patients were available, and the relationship between BRMS1 expression and pathological data was further analyzed (Table S1). In 21 of the samples, H5, H20 and H22 are the only three samples that exhibited tumor invasion and metastasis before resection operation. Notably, all three samples exhibited remarkably down-regulated BRMS1 expression in tumor tissues, indicating the potential negative correlation between HCC metastasis and BRMS1 expression. Next, we assessed the BRMS1 expression levels in several HCC cell lines with different metastatic capacities. While Hep3B and Huh-7 were characterized as noninvasive HCC cell lines [29], [30], SK-Hep1 was derived from the ascites of a patient with liver cancer, and SK-Hep1 was widely used in experimental metastasis assays [31]. MHCC97L, MHCC97H and MHCCLM6 cells established through *in vivo* selection were demonstrated to exhibit increasing invasive and metastatic potential [32], [33]. Whole cell lysates from these HCC lines were subjected to anti-BRMS1 immunoblotting (Fig. 1B). BRMS1 expression was significantly higher in two non-metastatic cell lines (Hep3B and Huh-7) than in four other metastatic cell lines. Furthermore, there was a proportional decrease in the expression level of BRMS1 in MHCC-97L, MHCC-97H and MHCC-LM6 cells. Taken together, the results suggest that BRMS1 was significantly down-regulated in HCC tissues, and loss of BRMS1 expression might correlate with HCC metastasis.

### Expression of BRMS1 sensitized HCC cells to apoptosis

**Figure 2:**
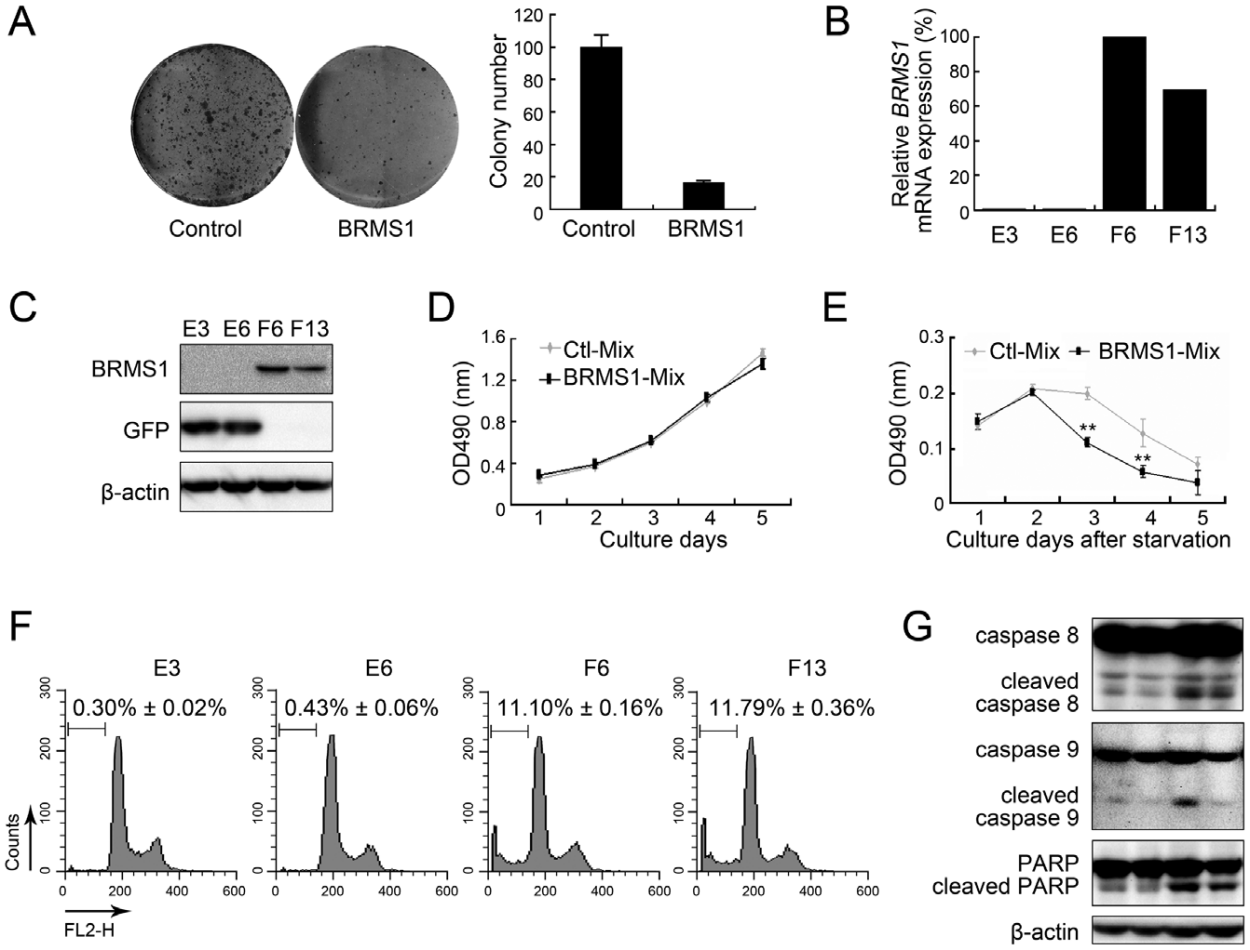
Effects of BRMS1 expression on SK-Hep1 cells. (A) A colony formation assay was performed on cells transiently transfected with vector control or a recombinant BRMS1 plasmid. Representative pictures are shown in the left panel and relative colony numbers are indicated in the histogram in the right panel. (B) Relative exogenous *BRMS1* mRNA expression in SK-Hep1 stable cell lines was quantifiedthrough qRT-PCR by normalization to *ACTB*. (C) Exogenous BRMS1 protein expression in stable cell lines was detected through western blotting analysis using anti-GFP and anti-BRMS1 antibodies. β-actin was used as a loading control. (D) Cell growth curves of mixed stable cell lines under normal culture conditions were calculated using the CCK8 assay. Values were indicated as mean ± SD, n = 5. (E) Cell growth curve of mixed stable lines under serum starvation. Values were indicated as mean ± SD, n = 5. (F) Cell apoptosis levels were determined by sub-G1 analysis using flow cytometry. Representative pictures are given and apoptotic cell ratios were presented as mean ± SD, n = 3. (G) Caspase 8, caspase 9 and PARP cleavage was investigated in stable cell lines undergoing anoikis through western blotting analysis using specific antibodies.

Based on the expression alternation of *BRMS1* in HCC, we further investigated the biological function of BRMS1 in regulating HCC cell activity. A colony formation assay using cells transfected with *BRMS1* was performed first, and the SK-Hep1 was selected due to its low level of endogenous *BRMS1* (Fig. 1B). Figure 2A shows that over-expression of *BRMS1* dramatically reduces the number of colonies that survive under G418 selection compared with the control group transfected with vector only, suggesting a suppressive role of BRMS1 over-expression in HCC cell viability. We then established stable BRMS1 transfectants in SK-Hep1 cells. Two BRMS1-positive clones (termed F6 and F13) and two control clones (termed E3 and E6) were identified through both qRT-PCR (Fig. 2B) and western blotting analysis (Fig. 2C). Next, to test whether BRMS1 expression affects cell growth under normal culture conditions, we generated two mixed clones (BRMS1-Mix and Ctl/control-Mix) by mixing stable cell clones in equal proportions and then recorded their growth daily. As shown in Fig. 2D, in a 5-day culture period, there was no obvious difference on the growth ratio between BRMS1-expressing cells and control cells. However, when cultured in the absence of serum, BRMS1-expressing cells displayed decreased cell number after 48 hours in culture compared to the control cells (Fig. 2E), suggesting that BRMS1 might sensitize cells to serum deprivation-induced apoptosis. Normal cells often die from anoikis as cells are detached from the extracellular matrix, while tumor cells are able to escape anoikis through oncogenic transformation [34]. To determine the involvement of BRMS1 in anoikis, cells were plated on a poly-HEMA-treated culture surface for 48 hours and then subjected to apoptosis analysis by measuring the sub-G1 population. As shown in Fig. 2F, both cell clones over-expressing BRMS1 displayed a significantly higher sub-G1 population (*P*<0.01) compared to the two control clones, indicating that increased apoptosis was induced by BRMS1 expression. Whole cell lysates from cells undergoing anoikis were then subjected to western blotting to measure caspase and PARP cleavage. As shown in Fig. 2G, both caspase 8 and caspase 9 cleavage was enhanced in two BRMS1-expressing clones, leading to enhanced PARP cleavage. All of these results implicate the contribution of BRMS1 in regulating the sensitivity of HCC cells to an apoptotic stimulus.

### Knockdown of BRMS1 protected HCC cells from apoptosis

**Figure 3:**
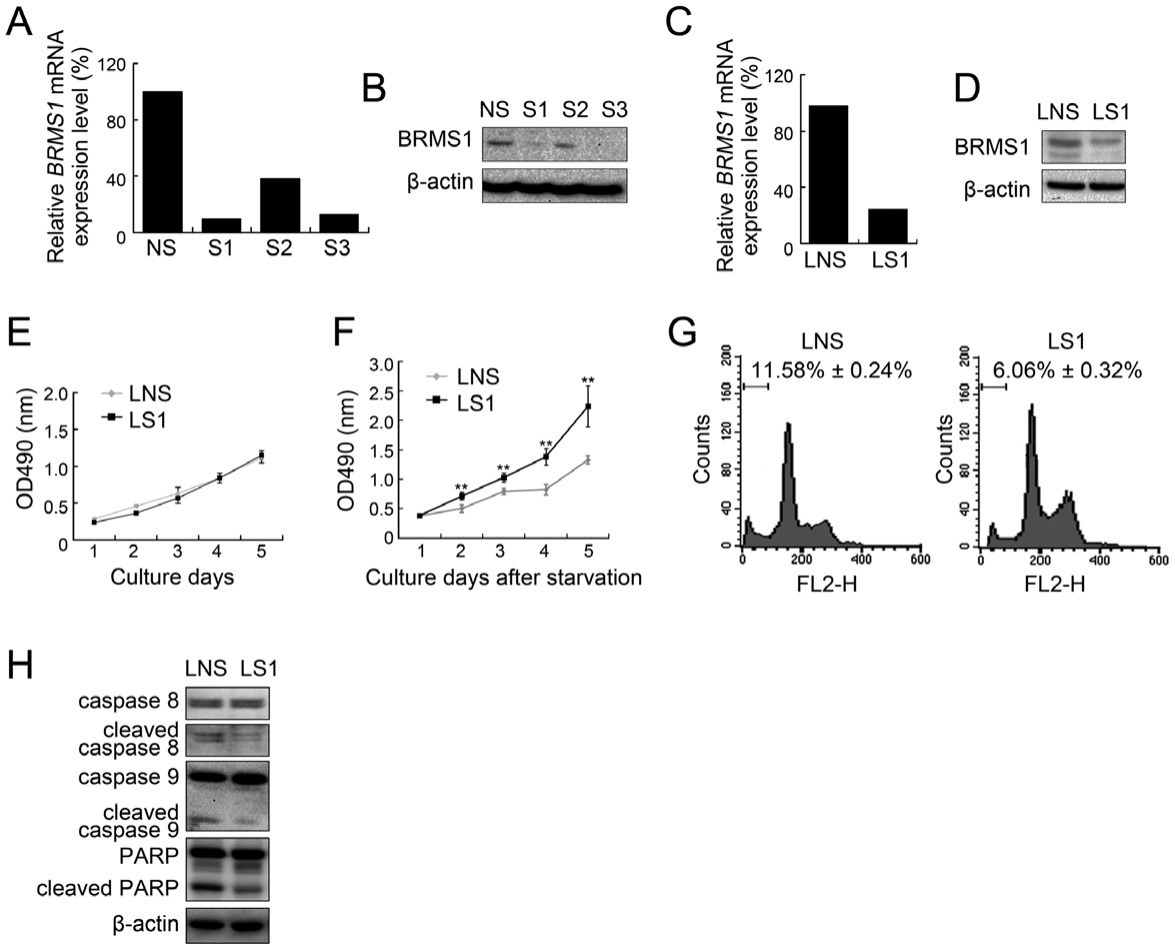
Effects of BRMS1 knockdown on Hep3B cells. Relative endogenous BRMS1 expression in Hep3B cells transfected with indicated siRNAs was analyzed by qRT-PCR (A) and western blotting analysis (B). Relative endogenous BRMS1 expression in Hep3B cells infected with the indicated lentivirus was analyzed by qRT-PCR (C) and western blotting (D). Cell growth curves of lentivirus-infected Hep3B cells under normal culture conditions (E) and serum deprivation (F). Values are indicated as mean ± SD, n = 5. (G) Cell apoptosis levels of lentivirus-infected Hep3B cells were determined through sub-G1 analysis. Representative flow cytometric pictures and apoptotic cell ratios are presented as mean ± SD, n = 3. (H) Caspase 8, caspase 9 and PARP cleavage was investigated in lentivirus-infected Hep3B cells undergoing anoikis.

To further confirm the correlation between BRMS1 and apoptosis in HCC, we investigated the role of endogenous BRMS1. We selected a HCC cell line with relatively high endogenous BRMS1 expression, Hep3B (Fig. 1B). Three small interfering RNAs targeting *BRMS1* mRNA, siRNA-1(S1), siRNA-2(S2) and siRNA-3(S3), as well as a non-specific control siRNA (NS), were designed and their effects on silencing *BRMS1* expression in Hep3B cells were measured. Both quantitative real-time PCR (qRT-PCR) and western blotting analysis showed that S1 and S3, but not S2, were able to suppress *BRMS1* expression at both mRNA and protein levels compared to NS (Fig. 3A, 3B). To establish stable cell lines, S1 was modified and further introduced into a lentivirus-based expression construct to generate the LS1 lentivirus. Control lentivirus (LNS) carrying NS was also generated. As shown in Fig. 3C and 3D, endogenous *BRMS1* expression at both mRNA and protein levels in Hep3B cells was effectively knocked down by LS1. Next, a cell growth assay was performed. Consistent with previous findings, while there is no detectable difference in cell growth ratios between cells infected with LS1 and control cells under normal culture conditions (Fig. 3E), cells infected with LS1 grew significantly faster than control cells after serum deprivation (Fig. 3F), implying that knockdown of BRMS1 might help cell grow under serum starvation. To confirm this hypothesis, we performed an anoikis assay, and consistent differences were observed. As shown in Fig. 3G, flow cytometric analysis revealed a significant decrease (*P*<0.01) in the percentage of the sub-G1 population in cells infected with LS1 compared to control cells. Moreover, both caspase 8 and caspase 9 cleavage was suppressed in cells with LS1 infection, and downstream PARP cleavage was also consequently suppressed (Fig. 3H). Therefore, consistent with results from BRMS1-expressing stable lines, knocking down endogenous BRMS1 expression protected HCC cells from apoptotic cell death.

### OPN is involved in BRMS1-regulated cell apoptosis

**Figure 4:**
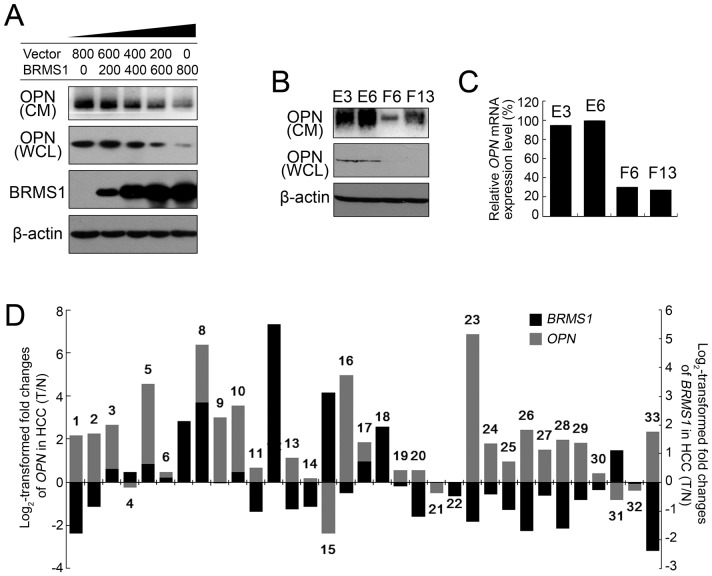
BRMS1 suppressed OPN expression in HCC cells. (A) Endogenous OPN expression in SK-Hep1 cells transfected with a gradient of doses of exogenous BRMS1 (ng/well) was investigated by western blotting using an anti-OPN antibody. Whole cell lysates (WCL) and conditioned medium (CM) were subjected to anti-OPN immunoblotting to detect intracellular OPN and secreted OPN, respectively. β-actin was used as a loading control. (B) Intracellular OPN and secreted OPN levels in SK-Hep1 stable cell lines were investigated by western blotting. (C) *OPN* mRNA expression levels in SK-Hep1 stable lines were investigated through qRT-PCR. Results are representative of independent experiments with consistent results. (D) Endogenous *BRMS1* (black box) and *OPN* (gray box) mRNA expression levels were analyzed in 33 HCC tumor tissues (No.1–33) and corresponding non-tumorous tissues through qRT-PCR and the 2^−ΔΔCt^ method as mentioned in Materials and Methods. A positive log_2_-transformed fold change value indicates higher expression levels in tumorous specimens compared to the non-tumorous specimens while a negative value indicates decreased relative mRNA levels.

Previous studies of BRMS1 in other tumor models revealed a transcriptional regulation role of BRMS1 in OPN expression [17]. It is therefore interesting to investigate whether BRMS1 is capable of regulating OPN in HCC cells. To address this question, we first investigated OPN expression changes in cells that were transiently transfected with BRMS1. As shown in Fig. 4A, compared with vector-only transfectants, *BRMS1* transfectants exhibited a dose-dependent suppression in OPN protein levels in both whole cell lysates (WCL) and conditioned medium (CM). In SK-Hep1 clones F6 and F13 stably expressing BRMS1, OPN levels in WCL and CM were also strikingly suppressed in comparison with control clones E3 and E6 (Fig. 4B). Consistently, *OPN* mRNA expression level in F6 and F13 was remarkably reduced compared to that in E3 and E6 (Fig. 4C), indicating the transcriptional regulation role of BRMS1 as a suppressor of OPN expression in HCC cells. To demonstrate the regulation relationship between BRMS1 and OPN at the tissue level, we further collected cDNA samples from the other 33 paired HCC tumor specimens to investigate the expression levels of both BRMS1 and OPN. Figure 4D shows the log_2_-transformed fold changes of *BRMS1* and *OPN* mRNA expression ratio of T/N (Tumor/Non-tumorous tissue). While *BRMS1* expression exhibited a consistent down-regulated pattern, *OPN* expression was significantly up-regulated in HCC tissues compared to non-tumorous tissues. More importantly, a negative correlation (*P* = 0.021, Fisher's exact test) between *BRMS1* and *OPN* mRNA expression was identified (Table 1). All of these data strongly suggested that BRMS1 negatively regulates OPN in HCC.

**Table 1:** Relationship between BRMS1 and OPN mRNA expression in HCC specimens.

HCC specimens		*BRMS1* down-regulation		positive ratio (n = 33)	*P*
		+	−		
*OPN* up-regulation	+	13	10	56.62%	0.021
	−	1	9	10.00%	

The relationship between *BRMS1* mRNA expression level and *OPN* mRNA expression level in 33 paired HCC tissues was analyzed by Fisher's exact test.

**Figure 5:**
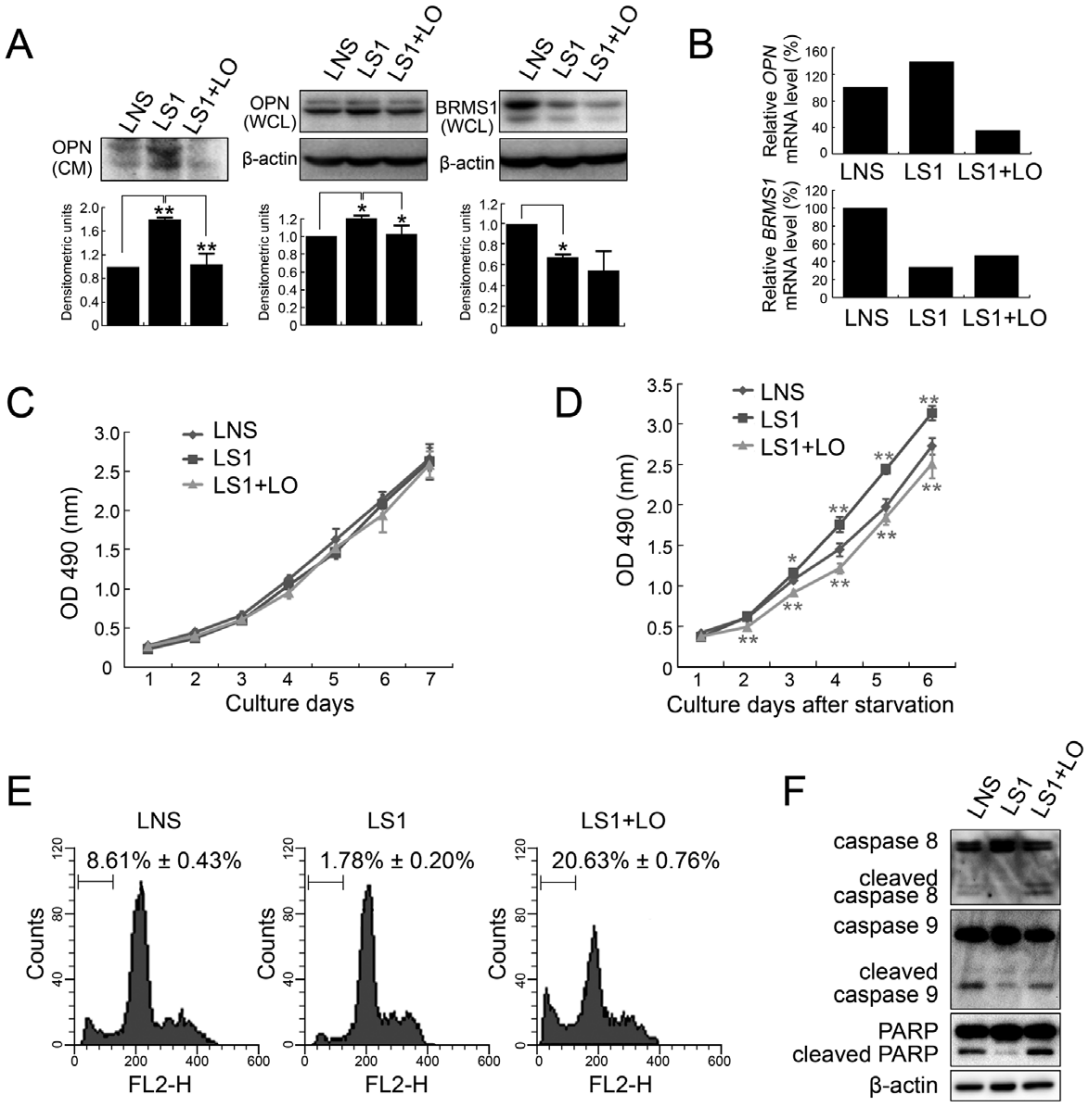
Effects of additional OPN knockdown on Hep3B cells. (A) Relative BRMS1 and OPN expressions in Hep3B cells infected with LNS, LS1 and LS1+LO were analyzed by western blotting. Representative pictures are shown and densitometric values are given in mean ± SD, n = 3. Both WCL and CM of cells were subjected to anti-OPN immunoblotting. (B) Relative *BRMS1* and *OPN* mRNA expressions in Hep3B cells were analyzed through qRT-PCR. Results are representative of independent experiments with consistent results. Cell growth curves of Hep3B infected with LNS, LS1 and LS1+LO were recorded under normal culture conditions (C) and serum deprivation (D). Values are indicated as mean ± SD, n = 5. (E) Cell apoptosis levels of lentivirus-infected cells were determined through sub-G1 analysis. Representative flow cytometric pictures are given and apoptotic cell ratios are presented as mean ± SD, n = 3. (F) Caspase 8, caspase 9 and PARP cleavage was detected in indicated Hep3B cell lines undergoing anoikis.

OPN is an important survival factor under many stress conditions [35]. Thus, we hypothesized that suppression of OPN expression might contribute to BRMS1-regulated cell apoptosis. To test this hypothesis, we generated another Hep3B cell line through infection with both LS1 and another lentivirus carrying effective shRNA (LO) targeting human *OPN* mRNA according to a previous report [36]. As shown in Fig. 5A and 5B, knockdown of endogenous BRMS1 alone up-regulated endogenous OPN expression levels at both mRNA and protein levels, providing additional evidence for the transcriptional suppression of OPN expression by BRMS1. However, infected cells with additional LO lentivirus (LS1+LO) successfully reversed *OPN* mRNA levels (Fig. 5B), and endogenous OPN protein expression in both WCL and CM was knocked down consistently (Fig. 5A). Similarly, cell growth curves were recorded under normal culture conditions and in the absence of serum. Additional LO infection exerted no obvious effect on cell growth under normal culture conditions (Fig. 5C); however, after serum deprivation, knockdown of endogenous OPN expression (LS1+LO) significantly eliminated cell growth advantages induced by BRMS1 silencing (LS1) (Fig. 5D). Next, we investigated cell apoptosis levels under anoikis conditions. Sub-G1 measurement for apoptosis analysis showed that while knocking down endogenous BRMS1 alone protected cells from apoptosis, additional silencing of OPN (LS1+LO) successfully reversed this effect, leading to a significant increase (*P*<0.01) in cell apoptosis (Fig. 5E). Western blotting analysis provided consistent evidence that cleavage of caspase 8, caspase 9 and downstream PARP was enhanced again after double silencing of *BRMS1* and *OPN* (Fig. 5F). Taken together, OPN is not only under the transcriptional control of BRMS1, but it also contributes to BRMS1-regulated cell apoptosis.

## Discussion

BRMS1 was initially identified in the chromosome region 11q13-q14, which exhibits a high frequency of deletion in late-stage, metastatic breast carcinomas patients [2]. Further studies showed that higher BRMS1 expression correlates with a better prognosis of breast carcinomas patients, and BRMS1 is active in suppressing breast carcinoma metastasis both *in vitro* and *in vivo* [3], [11]. More importantly, the tumor suppressor role of BRMS1 is not only limited to breast carcinomas. It has been demonstrated that BRMS1 is also involved in regulating the development of many other tumor types, such as melanoma, ovarian carcinoma and non-small lung cancer, among others [reviewed in [37]]. However, the role of BRMS1 in human HCC remains unclear.

Here, we reported for the first time that the expression of BRMS1 changes in HCC. Western blotting analysis of 31 paired HCC tissue samples revealed a significantly down-regulated expression pattern of BRMS1 in HCC (Fig. 1A). Promoter analysis of the *BRMS1* gene indicated that epigenetic silencing contributes to the reduction or loss of BRMS1 expression in breast carcinoma and non-small-cell lung cancer [38], [39]. Whether the same mechanism accounts for down-regulated HCC-associated BRMS1 expression requires further studies on the *BRMS1* promoter in HCC. By analyzing the clinico-pathological data from 21 specimens, we found that all three patients with tumor invasion and metastasis exhibited remarkably down-regulated BRMS1 expression. In addition, an obvious decrease in BRMS1 levels was found in four metastatic cell lines (SK-Hep1, MHCC-97L, MHCC-97H and MHCC-LM6) compared to the non-metastatic Hep3B and Huh-7 cell lines (Fig. 1B). Thus, reduced expression of BRMS1 might be predicative of increased metastatic activity in HCC cells. Although statistical analysis of the pathological data regarding BRMS1 expression in 21 HCC cases revealed no significant difference (Table S1), we have observed a potential relationship between BRMS1 expression and hepatitis virus infection. In patients with hepatitis, 53.33% exhibited down-regulated BRMS1 expression, while 83.33% of patients without hepatitis showed decreased BRMS1 expression. Moreover, while 55.56% HBsAg positive patients exhibited down-regulated BRMS1 expression, all HBsAg negative patients exhibited decreased BRMS1 expression. Viral infection has been shown to play a role in whole-genome epigenetic alteration [reviewed in [40]] and it has been reported that hypomethylation of specific chromosome regions was associated with hepatitis B virus integration [41]. We thus hypothesize that hepatitis virus infection may play a role in the induction of BRMS1 expression.

A successful metastasis event involves many critical steps. Generally, tumor cells first invade from the primary tumor site, then enter the circulation system and survive during the transportation, arrest and invade into the secondary site, and finally proliferate and grow at the new site [reviewed in [42]]. Tumor metastasis suppressor genes, including BRMS1, can perform their functions through blocking any one (or more) of these steps; for example, they can sensitize cells to an apoptotic stimulus. In the breast carcinoma model, BRMS1 mainly functions in regulating cell apoptosis, gap junctional intercellular communication and cell invasion, but does not affect cell growth or cell adhesion [15], [16], [43]. To elucidate the biological function of BRMS1 in HCC, we also investigated several cellular behaviors after expression or knockdown of BRMS1. In SK-Hep1 cells expressing BRMS1, no significant difference was shown in the cell growth ratio (Fig. 2D), cell cycle distribution and cell adhesion ability on fibronectin (data not shown) under normal culture conditions. Consistently, Hep3B cells exhibited no obvious difference in cell growth ability after endogenous BRMS1 was silenced (Fig. 3E). However, expression of BRMS1 sensitized SK-Hep1 cells to apoptosis triggered by serum starvation or matrix deprivation (anoikis) (Fig. 2E, 2F), whereas knockdown of BRMS1 rendered Hep3B cells resistant to apoptotic cell death (Fig. 3F, 3G). In addition, caspase 8 is a critical event in anoikis [44], and we have previously reported caspase 8 activation during anoikis of HCC cells [45]. Caspase 8 cross-talk with multiple other caspases (including caspase 9) was also shown in anoikis [46], [47]. Consistently, we found that BRMS1 affected both caspase 8 and caspase 9 cleavage in HCC cells undergoing anoikis, further demonstrating the functional link between BRMS1 and HCC cell apoptosis (Fig. 2G, 3H). Dysregulation of apoptosis signaling pathways are not only frequently observed in primary and metastatic HCC, but also contribute to the malignant phenotype of HCC. Altered expression of apoptosis-related genes and abnormal apoptotic levels in HCC cells are associated with cell invasion, tumor metastasis, tumor recurrence and poor prognosis [48], [49], [50]. Interfering with apoptosis signaling pathways has been shown to affect HCC growth and metastasis [51], [52], [53]. As we mentioned above, the ability of cancer cells to escape apoptosis is required for metastasis, especially anoikis resistance during transportation in the circulation system [reviewed in [54]]. Given that BRMS1 expression is able to impair apoptosis resistance under stress conditions (especially anoikis) in cultured HCC cells, we hypothesized that BRMS1 may exert activity in regulating HCC cell metastasis, but detailed information on this activity needs to be revealed by further experiments.

As a new component of mSin3a-HDAC complexes, BRMS1 was considered to carry out its metastasis suppressing function mainly through its indirect effect on regulation of gene transcription [13]. Several groups have conducted gene microarrays or 2D proteomic and mass spectrometry (MS) analysis to identify genes or proteins under the control of BRMS1 to delineate the molecular mechanism of BRMS1 [55], [56], [57], [58]. In our present work, we focused on one reported BRMS1-regulating gene, OPN, which has been identified as an unfavorable prognostic marker of HCC as well as an important gene that promotes HCC metastasis. Whether OPN is also regulated by BRMS1 in HCC was analyzed. Western blotting and qRT-PCR analysis of HCC cell lines either overexpressing BRMS1 or silencing BRMS1 (Fig. 4A, 4B, 4C, 5A 5B), demonstrated that BRMS1 was able to inhibit OPN expression in HCC. The effect of BRMS1 on OPN expression shows that it plays a suppressive role in regulating the transcriptional activity of the *OPN* gene promoter in HCC cells as demonstrated by dual luciferase assay *in vitro* (data not shown), which is consistent with previous reports in breast cancer [17]. More importantly, analysis of both *BRMS1* and *OPN* mRNA expression in paired HCC tissues revealed a negative correlation (Fig. 4D), highlighting a suppressive regulatory relationship between BRMS1 and OPN expression in HCC for the first time. A previous study identified TIP30 (30-kDa Tat-interacting protein) as a suppressive transcriptional regulator of OPN expression [59]. Through suppressing OPN expression, TIP30 suppresses cell adhesion and invasion, leading to reduced HCC growth and lung metastasis in a xenograft tumor model. Herein, we demonstrate that BRMS1 is another suppressive regulator of OPN in HCC, and BRMS1 may emerge as another potential HCC metastasis suppressor in further studies.

However, different from TIP30-OPN, BRMS1-OPN regulation exerted a co-operative effect on HCC cell apoptosis in our study. Using lentivirus-mediated RNA interference, we found that additional knockdown of OPN was able to rescue the anti-apoptotic effect induced by interfering endogenous BRMS1 expression, leading to elevated caspase cleavage (Fig. 5D, 5E). In breast carcinoma, Hedley *et al* generated consistent results showing that OPN contributes to BRMS1-mediated anchorage independent growth and hypoxia-induced apoptosis by utilizing MDA-MB-435 cell lines overexpressing BRMS1 alone and BRMS1 together with OPN [60]. In fact, OPN prevent cell apoptosis in multiple tumors and ultimately promotes tumor growth and metastasis [61], [62], [63]. In OPN-null mice, elevated cell apoptosis levels led to delayed papilloma development in chemically-induced tumor models [64]. Overexpression of OPN in pancreatic and fibrosarcoma cell lines suppressed cell apoptosis and promoted tumor growth and lung metastasis in a xenograft tumor model [65]. Consistent with our findings, knockdown of endogenous OPN using shRNA in HCC cells induced intrinsic apoptosis signaling and suppressed tumorigenicity and lung metastasis in xenograted nude mice, further demonstrating the anti-apoptosis activity of OPN in HCC [36]. Given that OPN is an effective therapeutic target for metastatic HCC, clarification of the signaling pathway of BRMS1-OPN regulation in the future would provide us a new and effective therapeutic approach for HCC.

## Materials and Methods

### Ethics Statement

This work was accomplished with the approval of the Medical Ethics Committee of School of Life Sciences, Fudan University. Written informed consent was obtained from all participants involved in this study.

### Tumor specimens

Fresh surgical specimens of HCC, including tumor tissues and the neighboring pathologically non-tumorous liver tissues, were obtained from liver cancer patients at Zhongshan Hospital, Shanghai, China. All of the samples were immediately frozen in liquid nitrogen after surgery and then stored at −80°C before further analysis.

### Cell culture and reagents

Cells were all cultured with Dulbecco's modified Eagle's medium (DMEM), supplemented with 10% fetal bovine serum at 37°C in 5% CO_2_-humidified atmosphere. All cell culture reagents were purchased from Gibco Laboratories (NY, USA). The rabbit polyclonal antibody against BRMS1 was purchased from Sigma (MO, USA), and the mouse monoclonal antibody against BRMS1 was purchased from Abcam (MA, USA). The mouse monoclonal antibodies against caspase 8, caspase 9 and rabbit polyclonal antibody against PARP were purchased from Cell Signaling Technology (MA, USA). The mouse monoclonal antibody against OPN was from R&D systems (MN, USA). The anti-β-actin monoclonal antibody and anti-GFP polyclonal antibody were from Sigma (MO, USA), and the anti-mouse/rabbit secondary antibodies were from Calbiochem (Germany).

### Western blotting

Protein samples were separated by SDS-PAGE and then transferred to PVDF membranes. After blocking, the membranes were incubated with specific antibodies against different proteins at 4°C overnight, followed by incubation with a horseradish peroxidase-conjugated secondary antibody. Immunoreactivity was visualized by enhanced chemiluminescence (GE healthcare, NJ, USA). All western blottings were performed at least twice, and the results are representative of independent experiments with consistent results.

### Transient transfection and selection of stable transfectants

The *BRMS1* coding sequence was cloned from human liver cDNA and then subcloned into the mammalian expression vector pEGFP-N2 containing GFP tag and a neomycin resistance gene for establishment of stable transfectants. Cells of 80% confluency were transfected with plasmids using Lipo2000 (Invitrogen, CA, USA) according to the manufacture's protocol. To screen stable cell lines, cells were seeded into new dishes 24 hours post-transfection and subjected to selection with 800 µg/mL G418 (Invitrogen, CA, USA) for approximately two weeks until single clones were visible. Independent colonies were isolated and subjected to western blotting using an anti-GFP antibody to select *BRMS1*-expressing colonies. Control colonies stably transfected with pEGFP-N2 empty vector were also generated in parallel.

### Colony formation assay

Cells at 80% confluency were transfected with pEGFP-N2 and pEGFP-BRMS1 separately. Cells were seeded into new 60 mm dishes 24 hours later at adensity of 1×10^5^/dish in triplicate. G418 was added into the medium 24 hours later. Colonies were identified by crystal violet staining after approximately 10–14 days in culture.

### Quantitative real-time PCR

Total RNA was extracted from tissues or cultured cells using Trizol reagent (Invitrogen, CA, USA), and 1–2 µg of RNA was used for reverse transcription using an oligo(dT) (Invitrogen, CA, USA) primer and Reverse Transcriptase (Takara, Japan). Quantitative real-time PCR (qRT-PCR) analysis was performed using the SYBR Green Supermix kit (Takara, Japan) with the Mastercycler ep realplex detection system (Eppendorf, Germany). Diluted cDNA was used in a 20 µl real-time PCR reaction in triplicate for each gene. Cycle parameters were 95°C for 5 min hot start and 45 cycles of 95°C for 5 sec, 60°C for 10 sec and 72°C for 20 sec. Blank controls with no cDNA templates were performed to rule out contamination. The specificity of the PCR product was confirmed by melting curve analysis. Primers for exogenous *BRMS1* were: forward seq., 5′-GTGTCCCCTCAGAAGAGAAAATCG-3′., reverse seq., 5′- CTCCTCGCCCTTGCTCACC-3′. Primers for endogenous *BRMS1* were: forward seq., 5′-ACTGAGTCAGCTGCGGTTGCGG-3′, reverse seq., 5′-AAGACCTGGAGCTGCCTCTGGCGTGC-3′. Primers for *OPN* were: forward seq., 5′-CCGAGGTGATAGTGTGGTTTATGG-3′, reverse seq., 5′- TGGACTGCTTGTGGCTGTGG-3′. Primers for *ACTB* were: forward seq., 5′-TACCACTGGCATCGTGATGGAC-3′, reverse seq., 5′-GATCTCCTTCTGCATCCTGTCG-3′. The expression levels of all genes were normalized to that of the house keeping gene *ACTB*. Relative gene expression levels were calculated by the formula 2^−ΔCt^, where ΔCt (Critical threshold) = Ct of genes of interest – Ct of *ACTB*. For analysis of HCC specimens, fold changes of gene expression levels in tumor specimens relative to corresponding non-tumorous specimens (T/N) were calculated by 2^−ΔΔCt^ method as previously described [45] and transformed to log_2_, where ΔΔCt = ΔCt tumor – ΔCt non-tumorous. The thresholds of a 1.5-fold change and a 0.5-fold change were set for identifying a significant increase and decrease in gene expression, respectively.

### Cell proliferation assay

Cells were plated at a density of 1500 cells/well in 96-well plates with complete medium. In the serum deprivation assay, culture medium was replaced by DMEM 24 hours after cell plating. The CCK8 assay was used to detect cell proliferation. Briefly, CCK8 (Dojindo, Japan) was added to DMEM to a final concentration of 0.5 mg/mL. After the culture medium was replaced by CCK8-containing medium, cells were then kept at 37°C for 4 hours. Results were obtained by measuring the absorbance at a wavelength of 490 nm read by a microtiter reader (Bio-RAD, CA, USA). The cell growth curve was calculated using the absorbance values (n = 6).

### Anoikis assay

Cells in normal culture were harvested by trypsinization and re-suspended in complete medium. Suspended cells were replated on poly-HEMA-coated (10 mg/mL, Sigma, MO, USA) dishes for the indicated amount of time before harvest.

### Flow cytometry analysis

For sub-G1 analysis, cells were harvested and resuspended in 70% ice-cold ethanol for fixation overnight. DNA was stained with propidium iodide (50 µg/mL) and treated with RNase (100 µg/mL) before analysis by FACSCalibur (BD Biosciences, CA, USA). The apoptotic cell population corresponds to cells in sub-G1 phase. Results are representative of three independent experiments with triplicate samples for each condition.

### siRNA screen and lentivirus infection

*BRMS1* siRNA1 (S1; sense seq.: 5′-GGAAUAAGUACGAAUGUGATT-3′), siRNA2 (S2; sense seq.: 5′-GGACUGGACAGCCAUCAAATT-3′), siRNA3 (S3; sense seq.:5′-GAAGACAGCCGAAGUCAAATT-3′) and a nonsilencing control were designed and synthesized at Genechem (Shanghai, China). Hep3B cells were transfected with siRNA targeted to *BRMS1* as well as NS siRNA using lipo2000 (Invitrogen, CA, USA). Quantification of *BRMS1* mRNA and protein were performed through qRT-PCR and western blotting analysis. Because siRNA S1 was the most effective, it was further cloned, along with the NS control, into a shRNA vector. The corresponding lentiviral particles for siRNA S1 and NS were packaged at Genechem and designated as LS1 and LNS, respectively. Lentiviruses carrying effective shRNA targeting OPN were purchased from Genechem and designated as LO. Hep3B cells cultured in 12-well plates were infected with LNS and LS1 at a multiplicity of infection (MOI) of 20 for each lentivirus. Silencing effects were confirmed by both qRT-PCR and western blotting analysis 72 hours after infection. LS1+LO cell lines were produced by infecting Hep3B cells with equal concentrations of two types of lentivirus at a MOI of 10 for each lentivirus.

### Statistical analysis

Comparisons of quantitative data were analyzed by Student's *t*-test. Categorical data were analyzed by Fisher's exact test. We considered P<0.05 to be different (*) and P<0.01 to be significant different (**).

## Supporting Information

Table S1 **Association analysis of BRMS1 expression and clinico-pathological data of HCC patients.** The relationship between clinico-pathological data of HCC patients and BRMS1 protein expression level was analyzed by Fisher's exact test.
(DOC)

## References

[1] Stafford LJ, Vaidya KS, Welch DR (2008) Metastasis suppressors genes in cancer. Int J Biochem Cell Biol 40: 874–891. 18280770 10.1016/j.biocel.2007.12.016

[2] Phillips KK, Welch DR, Miele ME, Lee JH, Wei LL, et al (1996) Suppression of MDA-MB-435 breast carcinoma cell metastasis following the introduction of human chromosome 11. Cancer Res 56: 1222–1227. 8640802

[3] Seraj MJ, Samant RS, Verderame MF, Welch DR (2000) Functional evidence for a novel human breast carcinoma metastasis suppressor, BRMS1, encoded at chromosome 11q13. Cancer Res 60: 2764–2769. 10850410

[4] Shevde LA, Samant RS, Goldberg SF, Sikaneta T, Alessandrini A, et al (2002) Suppression of human melanoma metastasis by the metastasis suppressor gene, BRMS1. Exp Cell Res 273: 229–239. 11822878 10.1006/excr.2001.5452

[5] Zhang S, Lin QD, Di W (2006) Suppression of human ovarian carcinoma metastasis by the metastasis-suppressor gene, BRMS1. Int J Gynecol Cancer 16: 522–531. 16681721 10.1111/j.1525-1438.2006.00547.x

[6] Jiang J, Xia M, Feng JB, Kong BH (2007) Inhibitory effect of breast cancer metastasis suppressor l gene on metastasis of human ovarian cancer cell in vitro and in vivo. Zhonghua Fu Chan Ke Za Zhi 42: 398–402. 17697602

[7] Smith PW, Liu Y, Siefert SA, Moskaluk CA, Petroni GR, et al (2009) Breast cancer metastasis suppressor 1 (BRMS1) suppresses metastasis and correlates with improved patient survival in non-small cell lung cancer. Cancer Lett 276: 196–203. 19111386 10.1016/j.canlet.2008.11.024 PMC4793277

[8] Cook LM, Cao X, Dowell AE, Debies MT, Edmonds MD, et al (2012) Ubiquitous Brms1 expression is critical for mammary carcinoma metastasis suppression via promotion of apoptosis. Clin Exp Metastasis 29: 315–325. 22241150 10.1007/s10585-012-9452-x PMC3660968

[9] Hicks DG, Yoder BJ, Short S, Tarr S, Prescott N, et al (2006) Loss of breast cancer metastasis suppressor 1 protein expression predicts reduced disease-free survival in subsets of breast cancer patients. Clin Cancer Res 12: 6702–6708. 17121889 10.1158/1078-0432.CCR-06-0635 PMC1661839

[10] Lombardi G, Di Cristofano C, Capodanno A, Iorio MC, Aretini P, et al (2007) High level of messenger RNA for BRMS1 in primary breast carcinomas is associated with poor prognosis. Int J Cancer 120: 1169–1178. 17163420 10.1002/ijc.22379

[11] Zhang Z, Yamashita H, Toyama T, Yamamoto Y, Kawasoe T, et al (2006) Reduced expression of the breast cancer metastasis suppressor 1 mRNA is correlated with poor progress in breast cancer. Clin Cancer Res 12: 6410–6414. 17085653 10.1158/1078-0432.CCR-06-1347

[12] Zainabadi K, Benyamini P, Chakrabarti R, Veena MS, Chandrasekharappa SC, et al (2005) A 700-kb physical and transcription map of the cervical cancer tumor suppressor gene locus on chromosome 11q13. Genomics 85: 704–714. 15885497 10.1016/j.ygeno.2005.02.014

[13] Meehan WJ, Samant RS, Hopper JE, Carrozza MJ, Shevde LA, et al (2004) Breast cancer metastasis suppressor 1 (BRMS1) forms complexes with retinoblastoma-binding protein 1 (RBP1) and the mSin3 histone deacetylase complex and represses transcription. J Biol Chem 279: 1562–1569. 14581478 10.1074/jbc.M307969200

[14] Cicek M, Fukuyama R, Welch DR, Sizemore N, Casey G (2005) Breast cancer metastasis suppressor 1 inhibits gene expression by targeting nuclear factor-kappaB activity. Cancer Res 65: 3586–3595. 15867352 10.1158/0008-5472.CAN-04-3139

[15] Saunders MM, Seraj MJ, Li Z, Zhou Z, Winter CR, et al (2001) Breast cancer metastatic potential correlates with a breakdown in homospecific and heterospecific gap junctional intercellular communication. Cancer Res 61: 1765–1767. 11280719

[16] Samant RS, Seraj MJ, Saunders MM, Sakamaki TS, Shevde LA, et al (2000) Analysis of mechanisms underlying BRMS1 suppression of metastasis. Clin Exp Metastasis 18: 683–693. 11827072 10.1023/a:1013124725690

[17] Samant RS, Clark DW, Fillmore RA, Cicek M, Metge BJ, et al (2007) Breast cancer metastasis suppressor 1 (BRMS1) inhibits osteopontin transcription by abrogating NF-kappaB activation. Mol Cancer 6: 6. 17227585 10.1186/1476-4598-6-6 PMC1796551

[18] Wai PY, Kuo PC (2008) Osteopontin: regulation in tumor metastasis. Cancer Metastasis Rev 27: 103–118. 18049863 10.1007/s10555-007-9104-9

[19] Takafuji V, Forgues M, Unsworth E, Goldsmith P, Wang XW (2007) An osteopontin fragment is essential for tumor cell invasion inhepatocellular carcinoma. Oncogene 26: 6361–6371. 17452979 10.1038/sj.onc.1210463

[20] Chen RX, Xia YH, Xue TC, Zhang H, Ye SL (2011) Down-regulation of osteopontin inhibits metastasis of hepatocellular carcinoma cells via a mechanism involving MMP-2 and uPA. Oncol Rep 25: 803–808. 21174062 10.3892/or.2010.1116

[21] Ye QH, Qin LX, Forgues M, He P, Kim JW, et al (2003) Predicting hepatitis B virus-positive metastatic hepatocellular carcinomas using gene expression profiling and supervised machine learning. Nat Med 9: 416–423. 12640447 10.1038/nm843

[22] Pan HW, Ou YH, Peng SY, Liu SH, Lai PL, et al (2003) Overexpression of osteopontin is associated with intrahepatic metastasis, early recurrence, and poorer prognosis of surgically resected hepatocellular carcinoma. Cancer 98: 119–127. 12833464 10.1002/cncr.11487

[23] Kim J, Ki SS, Lee SD, Han CJ, Kim YC, et al (2006) Elevated plasma osteopontin levels in patients with hepatocellular carcinoma. Am J Gastroenterol 101: 2051–2059. 16848813 10.1111/j.1572-0241.2006.00679.x

[24] Huang H, Zhang XF, Zhou HJ, Xue YH, Dong QZ, et al (2010) Expression and prognostic significance of osteopontin and caspase-3 in hepatocellular carcinoma patients after curative resection. Cancer Sci 101: 1314–1319. 20345480 10.1111/j.1349-7006.2010.01524.x PMC11159602

[25] Zhang CH, Xu GL, Jia WD, Ge YS, Li JS, et al (2012) Prognostic significance of osteopontin in hepatocellular carcinoma: a meta-analysis. Int J Cancer 130: 2685–2692. 21780114 10.1002/ijc.26301

[26] Zhang H, Ye QH, Ren N, Zhao L, Wang YF, et al (2006) The prognostic significance of preoperative plasma levels of osteopontin in patients with hepatocellular carcinoma. J Cancer Res Clin Oncol 132: 709–717. 16786357 10.1007/s00432-006-0119-3 PMC12161026

[27] Yang GH, Fan J, Xu Y, Qiu SJ, Yang XR, et al (2008) Osteopontin combined with CD44, a novel prognostic biomarker for patients with hepatocellular carcinoma undergoing curative resection. Oncologist 13: 1155–1165. 18997126 10.1634/theoncologist.2008-0081

[28] Yu MC, Lee YS, Lin SE, Wu HY, Chen TC, et al (2011) Recurrence and Poor Prognosis Following Resection of Small Hepatitis B-Related Hepatocellular Carcinoma Lesions Are Associated with Aberrant Tumor Expression Profiles of Glypican 3 and Osteopontin. Ann Surg Oncol Epub ahead of print. 10.1245/s10434-011-1946-2 21822558

[29] Giannelli G, Bergamini C, Fransvea E, Marinosci F, Quaranta V, et al (2001) Human hepatocellular carcinoma (HCC) cells require both alpha3beta1 integrin and matrix metalloproteinases activity for migration and invasion. Lab Invest 81: 613–627. 11304581 10.1038/labinvest.3780270

[30] Nakabayashi H, Taketa K, Miyano K, Yamane T, Sato J (1982) Growth of human hepatoma cells lines with differentiated functions in chemically defined medium. Cancer Res 42: 3858–3863. 6286115

[31] Huang CS, Liao JW, Hu ML (2008) Lycopene inhibits experimental metastasis of human hepatoma SK-Hep-1 cells in athymic nude mice. J Nutr 138: 538–543. 18287363 10.1093/jn/138.3.538

[32] Li Y, Tang ZY, Ye SL, Liu YK, Chen J, et al (2001) Establishment of cell clones with different metastatic potential from the metastatic hepatocellular carcinoma cell line HCC97. World J Gastroenterol 7: 630–636. 11819844 10.3748/wjg.v7.i5.630 PMC4695564

[33] Xue YH, Zhang XF, Dong QZ, Sun J, Dai C, et al (2010) Thrombin is a therapeutic target for metastatic osteopontin-positive hepatocellular carcinoma. Hepatology 52: 2012–2022. 20890897 10.1002/hep.23942

[34] Frisch SM, Francis H (1994) Disruption of epithelial cell-matrix interactions induces apoptosis. J Cell Biol 124: 619–626. 8106557 10.1083/jcb.124.4.619 PMC2119917

[35] Malyankar UM, Scatena M, Suchland KL, Yun TJ, Clark EA, et al (2000) Osteoprotegerin is an alpha vbeta 3-induced, NF-kappa B-dependent survival factor for endothelial cells. J Biol Chem 275: 20959–20962. 10811631 10.1074/jbc.C000290200

[36] Zhao J, Dong L, Lu B, Wu G, Xu D, et al (2008) Down-regulation of osteopontin suppresses growth and metastasis of hepatocellular carcinoma via induction of apoptosis. Gastroenterology 135: 956–968. 18555021 10.1053/j.gastro.2008.05.025

[37] Hurst DR, Welch DR (2011) Metastasis suppressor genes at the interface between the environment and tumor cell growth. Int Rev Cell Mol Biol 286: 107–180. 21199781 10.1016/B978-0-12-385859-7.00003-3 PMC3575029

[38] Metge BJ, Frost AR, King JA, Dyess DL, Welch DR, et al (2008) Epigenetic silencing contributes to the loss of BRMS1 expression in breast cancer. Clin Exp Metastasis 25: 753–763. 18566899 10.1007/s10585-008-9187-x PMC2763604

[39] Yang J, Shen Y, Liu B, Tong Y (2011) Promoter methylation of BRMS1 correlates with smoking history and poor survival in non-small cell lung cancer patients. Lung Cancer 74: 305–309. 21726917 10.1016/j.lungcan.2011.03.002

[40] Li HP, Leu YW, Chang YS (2005) Epigenetic changes in virus-associated human cancers. Cell Res 15: 262–271. 15857581 10.1038/sj.cr.7290295

[41] Nagai H, Baba M, Konishi N, Kim YS, Nogami M, et al (1999) Isolation of NotI clusters hypomethylated in HBV-integrated hepatocellular carcinomas by two-dimensional electrophoresis. DNA Res 6: 219–225. 10492168 10.1093/dnares/6.4.219

[42] Gupta GP, Massague J (2006) Cancer metastasis: building a framework. Cell 127: 679–695. 17110329 10.1016/j.cell.2006.11.001

[43] Kapoor P, Saunders MM, Li Z, Zhou Z, Sheaffer N, et al (2004) Breast cancer metastatic potential: correlation with increased heterotypic gap junctional intercellular communication between breast cancer cells and osteoblastic cells. Int J Cancer 111: 693–697. 15252837 10.1002/ijc.20318

[44] Rytömaa M, Martins LM, Downward J (1999) Involvement of FADD and caspase-8 signalling in detachment-induced apoptosis. Curr Biol 9: 1043–1046. 10508619 10.1016/s0960-9822(99)80454-0

[45] Wu Y, Zuo J, Ji G, Saiyin H, Liu X, et al (2009) Proapoptotic function of integrin beta(3) in human hepatocellular carcinoma cells. Clin Cancer Res 15: 60–69. 19118033 10.1158/1078-0432.CCR-08-1028 PMC3658616

[46] Jiang C, Wang Z, Ganther H, Lu J (2001) Caspases as key executors of methyl selenium-induced apoptosis (anoikis) of DU-145 prostate cancer cells. Cancer Res 61: 3062–3070. 11306488

[47] Aguzzi MS, Giampietri C, De Marchis F, Padula F, Gaeta R, et al (2004) RGDS peptide induces caspase 8 and caspase 9 activation in human endothelial cells. Blood 103: 4180–4187. 14982875 10.1182/blood-2003-06-2144

[48] Ito Y, Monden M, Takeda T, Eguchi H, Umeshita K, et al (2000) The status of Fas and Fas ligand expression can predict recurrence of hepatocellular carcinoma. Br J Cancer 82: 1211–1217. 10735508 10.1054/bjoc.1999.1065 PMC2363358

[49] Ito Y, Matsuura N, Sakon M, Takeda T, Umeshita K, et al (1999) Both cell proliferation and apoptosis significantly predict shortened disease-free survival in hepatocellular carcinoma. Br J Cancer 81: 747–751. 10574266 10.1038/sj.bjc.6690758 PMC2362905

[50] Shi YH, Ding WX, Zhou J, He JY, Xu Y, et al (2008) Expression of X-linked inhibitor-of-apoptosis protein in hepatocellular carcinoma promotes metastasis and tumor recurrence. Hepatology 48: 497–507. 18666224 10.1002/hep.22393 PMC2768766

[51] Strand S, Hofmann WJ, Hug H, Müller M, Otto G, et al (1996) Lymphocyte apoptosis induced by CD95 (APO-1/Fas) ligand-expressing tumor cells–a mechanism of immune evasion? Nat Med 2: 1316–1366. 8946836 10.1038/nm1296-1361

[52] Li WC, Ye SL, Sun RX, Liu YK, Tang ZY, et al (2006) Inhibition of growth and metastasis of human hepatocellular carcinoma by antisense oligonucleotide targeting signal transducer and activator of transcription 3. Clin Cancer Res 12: 7140–7148. 17145839 10.1158/1078-0432.CCR-06-0484

[53] Hu H, Li Z, Chen J, Wang D, Ma J, et al (2010) P16 reactivation induces anoikis and exhibits antitumour potency by downregulating Akt/survivin signalling in hepatocellular carcinoma cells. Gut 60: 710–721. 20971978 10.1136/gut.2010.220020

[54] Simpson CD, Anyiwe K, Schimmer AD (2008) Anoikis resistance and tumor metastasis. Cancer Lett 272: 177–185. 18579285 10.1016/j.canlet.2008.05.029

[55] Cicek M, Samant RS, Kinter M, Welch DR, Casey G (2004) Identification of metastasis-associated proteins through protein analysis of metastatic MDA-MB-435 and metastasis-suppressed BRMS1 transfected-MDA-MB-435 cells. Clin Exp Metastasis 21: 149–157. 15168732 10.1023/b:clin.0000024729.19084.f0

[56] Rivera J, Megias D, Bravo J (2007) Proteomics-based strategy to delineate the molecular mechanisms of the metastasis suppressor gene BRMS1. J Proteome Res 6: 4006–4018. 17854218 10.1021/pr0703167

[57] Champine PJ, Michaelson J, Weimer BC, Welch DR, DeWald DB (2007) Microarray analysis reveals potential mechanisms of BRMS1-mediated metastasis suppression. Clin Exp Metastasis 24: 551–565. 17896182 10.1007/s10585-007-9092-8 PMC2214901

[58] Edmonds MD, Hurst DR, Vaidya KS, Stafford LJ, Chen D, et al (2009) Breast cancer metastasis suppressor 1 coordinately regulates metastasis-associated microRNA expression. Int J Cancer 125: 1778–1785. 19585508 10.1002/ijc.24616 PMC2749950

[59] Zhao J, Lu B, Xu H, Tong X, Wu G, et al (2008) Thirty-kilodalton Tat-interacting protein suppresses tumor metastasis by inhibition of osteopontin transcription in humanhepatocellular carcinoma. Hepatology 48: 265–275. 18537194 10.1002/hep.22280

[60] Hedley BD, Welch DR, Allan AL, Al-Katib W, Dales DW, et al (2008) Downregulation of osteopontin contributes to metastasis suppression by breast cancer metastasis suppressor 1. Int J Cancer 123: 526–534. 18470911 10.1002/ijc.23542 PMC13445066

[61] Graessmann M, Berg B, Fuchs B, Klein A, Graessmann A (2007) Chemotherapy resistance of mouse WAP-SVT/t breast cancer cells is mediated by osteopontin, inhibiting apoptosis downstream of caspase-3. Oncogene 26: 2840–2850. 17160024 10.1038/sj.onc.1210096

[62] Lee JL, Wang MJ, Sudhir PR, Chen GD, Chi CW, et al (2007) Osteopontin promotes integrin activation through outside-in and inside-out mechanisms: OPN-CD44V interaction enhances survival in gastrointestinal cancer cells. Cancer Res 67: 2089–2097. 17332338 10.1158/0008-5472.CAN-06-3625

[63] Song G, Cai QF, Mao YB, Ming YL, Bao SD, et al (2008) Osteopontin promotes ovarian cancer progression and cell survival and increases HIF-1alpha expression through the PI3-K/Akt pathway. Cancer Sci 99: 1901–1907. 19016748 10.1111/j.1349-7006.2008.00911.x PMC11158665

[64] Hsieh YH, Juliana MM, Hicks PH, Feng G, Elmets C, et al (2006) Papilloma development is delayed in osteopontin-null mice: implicating an antiapoptosis role for osteopontin. Cancer Res 66: 7119–7127. 16849558 10.1158/0008-5472.CAN-06-1002

[65] Courter D, Cao H, Kwok S, Kong C, Banh A, et al (2010) The RGD domain of human osteopontin promotes tumor growth and metastasis through activation of survival pathways. PLoS One 5: e9633. 20224789 10.1371/journal.pone.0009633 PMC2835762

