# Supplementary material for: Breast Cancer Metastasis Suppressor 1 Regulates Hepatocellular Carcinoma Cell Apoptosis via Suppressing Osteopontin Expression
Source: PLoS One. 2012 Aug 21;7(8):e42976. doi: 10.1371/journal.pone.0042976 (PMC3424258; doi:10.1371/journal.pone.0042976)
Supplement: Table S1 — Association analysis of BRMS1 expression and clinico-pathological data of HCC patients. The relationship between clinico-pathological data of HCC patients and BRMS1 protein expression level was analyzed by Fisher's exact test. (DOC) [file pone.0042976.s001.doc]

***Table S1. Association analysis of BRMS1 expression and clinico-pathological data of HCC patients***.

| Pathological characteristics | | *BRMS1* down regulation | | | *P* |
| --- | --- | --- | --- | --- | --- |
|  | | + | - | Positive ratio |  |
| Sex | Male | 12 | 7 | 63.16% | > 0.05 |
|  | Female | 1 | 1 | 50.00% |  |
| Age | ≤45 | 5 | 2 | 71.43% | > 0.05 |
|  | ＞45 | 8 | 6 | 57.14% |  |
| Hepatitis | (+) | 8 | 7 | 53.33% | > 0.05 |
|  | (-) | 5 | 1 | 83.33% |  |
| HBsAg | (+) | 10 | 8 | 55.56% | > 0.05 |
|  | (-) | 3 | 0 | 100.00% |  |
| AFP | (+) | 7 | 4 | 63.64% | > 0.05 |
|  | (-) | 6 | 4 | 60.00% |  |
| Tumor blot | ≥2 | 6 | 3 | 66.67% | > 0.05 |
|  | 1 | 7 | 5 | 58.33% |  |
| Tumor size | ＞5 cm | 8 | 5 | 61.54% | > 0.05 |
|  | ≤5 cm | 5 | 3 | 62.50% |  |
| Tumor invasion | (+) | 3 | 0 | 100.00% | > 0.05 |
|  | (-) | 10 | 8 | 55.56% |  |
| Differentiation | Grade I、II | 10 | 5 | 66.67% | > 0.05 |
|  | Grade III | 3 | 3 | 50.00% |  |
| Phase | 1 | 10 | 6 | 62.50% | > 0.05 |
|  | 2, 3 | 3 | 2 | 60.00% |  |

The relationship between different clinico-pathological data of HCC patients and BRMS1 protein expression level was analyzed by Fisher’s exact test.
